# Definitions of neonatal abstinence syndrome in clinical studies of mothers and infants: an expert literature review

**DOI:** 10.1038/s41372-020-00893-8

**Published:** 2021-01-29

**Authors:** Shahla M. Jilani, Chloe J. Jordan, Lauren M. Jansson, Jonathan M. Davis

**Affiliations:** 1 Office of the Assistant Secretary for Health, U.S. Department of Health and Human Services, Washington, DC USA; 2 Division of Extramural Research, National Institute on Drug Abuse, Rockville, MD USA; 3 Johns Hopkins University School of Medicine, Department of Pediatrics, Center for Addiction and Pregnancy, Baltimore, MD USA; 4 Division of Newborn Medicine, Tufts Children’s Hospital, Boston, MA USA; 5 The Tufts Clinical and Translational Science Institute, Tufts University, Boston, MA USA

**Keywords:** Signs and symptoms, Scientific community

## Abstract

Neonatal abstinence syndrome (NAS) results from discontinuation of in utero exposures to opioids/substances. The rising incidence of NAS has prompted an increased need for accurate research and public health data. To examine how NAS has been defined in clinical studies of opioid-exposed mothers and infants, a review process was developed based on the RAND/UCLA Appropriateness Method, yielding 888 abstracts. Per inclusion criteria, 57 abstracts underwent full-text review. To define NAS, studies cited using modified versions of the Finnegan NAS scoring tool (*n* = 21; 37%), ICD-9/10 coding (*n* = 17; 30%), original Finnegan tool (*n* = 16; 28%), Eat Sleep Console (*n* = 3; 5%), and Lipsitz (*n* = 3; 5%) tools, (3 cited 2+ tools). Most studies utilized subjective NAS scoring/assessment algorithms and neonatal coding as key elements defining NAS. While most cited opioid exposure as integral to their inclusion criteria, 26% did not. These approaches highlight the need for a more refined and standardized definition of NAS.

## Introduction

More than 45 years have elapsed since the seminal work describing neonatal abstinence syndrome (NAS) as a disorder in infants due to withdrawal from opioids and other substances after in utero exposures [1]. Today, prenatal opioid exposure and NAS remain a significant public health problem. From 2004 to 2014, NAS incidence increased >5-fold from 2.8 to 14.4 per 1000 Medicaid-reimbursed births [2, 3] prompting a focus on improving public health reporting and surveillance. In 2019, the Council of State and Territorial Epidemiologists (CSTE) developed a NAS case definition to standardize surveillance measures throughout different jurisdictions (local and state) across the U.S. [4]. Using a tiered approach, this case definition focused on case identification based on clinical and administrative claims records and was designed to advance standardization of health care provider reporting.

Upstream of case reporting, the definition of NAS can vary significantly among providers and researchers. Historically, most clinical descriptions of NAS have included a constellation of signs and symptoms of withdrawal that may require pharmacologic intervention [5, 6]. NAS has alternatively been described in the literature as any signs/symptoms of withdrawal in substance-exposed infants, regardless of the need for medication treatment [7], or signs/symptoms of withdrawal in infants that do require medication treatment. Although most commonly conceptualized as withdrawal from opioids in exposed infants, other substance exposures and maternal/infant factors can affect NAS expression, further complicating criteria for a definition of NAS [8]. Often heterogeneously defined [9], NAS can connote opioid-exposed infants and/or exposure to other substances, demonstration of withdrawal signs/symptoms, and/or receiving pharmacotherapy for withdrawal. Notably, consistent/standard criteria defining NAS are lacking. This could have a significant impact on the accuracy and reproducibility of claims and/or scientific data, especially when using electronic medical record data.

Without evidence-based criteria or consensus guidelines on how to clinically define NAS, guidance that standardizes the clinical diagnosis is urgently needed. As part of an overarching effort to establish a standard clinical definition of NAS, the U.S. Department of Health and Human Services (HHS) has partnered with national maternal–child specialists to systemically examine this issue through an appropriateness study using the RAND/UCLA Appropriateness Method (RAM). Of the evidence-based approaches available for assessing clinical validity and appropriateness, RAM is unique in offering the opportunity to leverage broad expert input to address key knowledge gaps in the literature [10]. The first step of the RAM process is a critical literature review, which is the focus of this article.

The key objective of this RAM literature review was to identify criteria described in clinical studies to define NAS. In the absence of standard clinical criteria, an understanding of key criteria and/or methods that have been used to define NAS and identification of relevant gaps or inconsistencies may inform critical discussions on how to approach the development of standardized clinical criteria. This is essential for both clinical care and clinical trials involving infants affected by NAS. While the lack of standard definitions for NAS has been described previously in various degrees of scope and depth, to date there nonetheless remains a paucity of standardized clinical criteria. The results of this RAM literature review will ultimately be used to develop guidance by HHS in collaboration with experts nationwide in maternal and child health, on standardizing the clinical diagnosis of NAS/NOWS (neonatal opioid withdrawal syndrome) for use by the broader clinical and research communities.

## Methods

The RAM literature review has been used in numerous research studies, including to support national guidance on treating pregnant and parenting women with opioid use disorder [11]. Similarly, we set out to examine available literature in order to develop a synthesis of scientific evidence specific to this research question, distinct from statistical methods employed in meta-analyses. This RAM literature review was carried out between September 2019 and June 2020. A Federal Steering Committee (FSC) and Expert Panel (EP) were assembled to conduct this review. The FSC consisted of maternal–child health and policy experts from the following federal agencies: Office of the Assistant Secretary for Planning and Evaluation, Centers for Disease Control and Prevention, Health Resources and Services Administration, Indian Health Service, and the National Institutes of Health. The EP consisted of clinical and research specialists in maternal–child health from obstetrics and gynecology, addiction medicine, general and hospital pediatrics, developmental pediatrics, primary care, and neonatology.

### Keyword search terms

For design of the initial keyword search, the World Health Organization Guidelines for the Identification and Management of Substance Use and Substance Use Disorders in Pregnancy and the American Academy of Pediatrics Clinical Report: Neonatal Drug Withdrawal were referenced [12, 13]. Based on these two guideline documents, the EP generated a broad keyword search with the following terms: NAS and NOWS, clinical signs/symptoms/diagnosis, opioids, specific types of opioids (e.g., buprenorphine, methadone, morphine, fentanyl, oxycodone, heroin), infant/newborn, withdrawal, urine/meconium/umbilical cord/hair, and the most common assessment tools, such as: (1) the Finnegan NAS scoring system, (2) modified versions of the Finnegan, (3) the Eat, Sleep, Console (ESC) assessment tool, and (4) the Lipsitz Neonatal Drug Withdrawal Scoring System. Of note, during the full-text review phase of this process (see below), no additional assessment tools were identified as in use for defining/diagnosing NAS or NOWS. In September 2019, an environmental scan using these keywords was carried out to gain insight on the breadth of literature in this subject area. Although this preliminary scan yielded a high volume of abstracts (*n* = 2817; data not shown), there was lower specificity for the primary research question (identifying specific criteria used to define NAS). It is important to note that the keyword “opioids” was utilized to retrieve any occurrence of opioids in combination with the described terms. However, retrieved abstracts were found to demonstrate low topical relevance. Thus this term was then removed in order to develop an appropriately focused keyword search.

### Search design

Utilizing the results of the environmental scan to inform and tailor subsequent review of the literature, the scope of the search strategy was subsequently narrowed to specifically identify abstracts containing criteria used in the definition or description of NAS. In December 2019, a search was run in PubMed, Embase, Cochrane, and CINAHL. Specific search terms included (1) NAS; (2) NOWS; (3) urine, umbilical cord, and/or meconium and NAS or NOWS; and (4) criteria, criterion, definition, define, or diagnosis of NAS or NOWS. This combination of search terms included literature published worldwide in English without date limitations and candidate abstracts highly specific to the primary research question.

The role of non-opioid other or polysubstance exposure (e.g., nicotine, other licit and illicit psychotropic drugs) in the pathogenesis of NAS was also considered, but current evidence was unclear as to specific causal associations so these agents were not added to the keyword search. However, retrieved abstracts were not excluded if found to address the primary research question while including other substance exposure in the study design. In other words, abstracts not directly assessing opioid exposure or assessing non-opioid or other substance exposures were not excluded. Rather, these terms were not distinctly specified in the keyword search. Additionally, given variability/availability in toxicology testing practices across the country, this specific terminology was not added to the keyword search.

### Inclusion and exclusion criteria

**Fig. 1:**
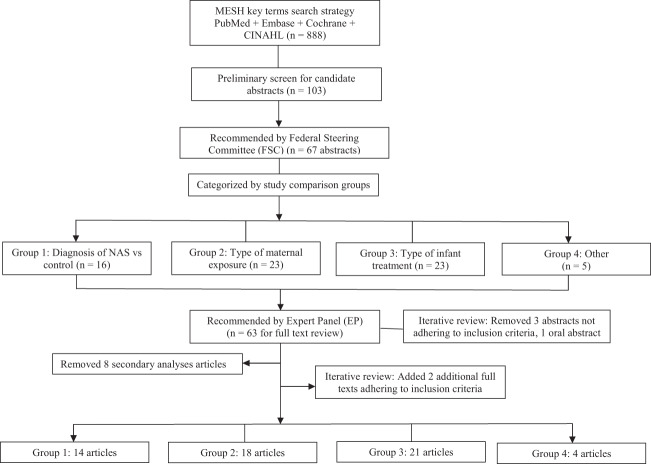
Flowchart summarizing overarching design and step-by-step methodology. After a preliminary screen for candidate abstracts, the FSC recommended 67 abstracts for further review. This recommended set was then independently reviewed by the EP, which selected 63 abstracts for full-text review; an iterative review confirmed removal of 3 abstracts as not fully adhering to inclusion criteria (and 1 oral abstract). Eight additional abstracts were removed as secondary analysis articles. Final iterative review of the full set of 888 resulted in 2 additional abstracts advanced for full-text review. Abstracts were categorized by study comparison groups to facilitate a more structured review process.

The focused keyword search yielded 888 abstracts after which duplicates, poster abstracts, animal studies, review articles, gray literature (white papers, government and academic reports not published by commercial publishers), and foreign language papers were removed (Fig. 1). More comprehensive reviews have previously described limitations in NAS literature; more specifically, noting a predominance of uncontrolled and nonrandomized studies with lower generalizability [14]. As a more focused review, key inclusion criteria for this review were targeted to randomized control trials, cohort trials, and case controls and/or the inclusion of a comparison/control group in the study design. Exclusion criteria included case reports, descriptive studies, observational, and other studies not designed to investigate outcomes between two distinct comparison groups, as a preliminary screen to promote inclusion of scientifically and analytically rigorous reports. It is noteworthy that the latter (studies not assessing distinct comparison groups) may offer descriptions of the NAS definition that could be contributory to this discussion. However, given the targeted scope of this literature review—identifying specific criteria used to define NAS—the presence of a non-NAS or other pertinent comparison group was specified as inclusion criteria essential to this primary research question.

A preliminary screen of the complete set based on the described criteria put forth 103 candidate abstracts representative of comparative studies with topical relevance, which were subsequently presented to the FSC for consideration. This preliminary screen was not an in-depth review, but rather, a broad overview scan to initiate the abstract review process. Abstracts then underwent in-depth review by members of the FSC who ultimately recommended 67 as matching to inclusion criteria. To ensure fidelity, an internal review was developed wherein a single reviewer independently examined recommendations for inclusion/removal of abstracts and articles. This approach helped to optimize internal consistency at each stage of the review process.

### Categorization of abstracts

The abstract inclusion process yielded a number of different types of comparative studies, presenting the need to sort abstracts for a more structured review by category. Accounting for individual study types, most abstracts sorted into three distinct types of study design and thus were categorized into comparison groups as follows: (1) studies focused on infants with a diagnosis of NAS vs those without NAS (this definition varied by study, typically described as non-opioid exposed or matched healthy infants), (2) studies designed to examine different types of maternal medications and/or exposures, and (3) studies investigating one infant treatment paradigm compared to another. Abstracts were divided into these three structured categories for subsequent full-text review based on the rationale that these different study types/points of focus may preferentially involve one assessment tool over another (see “Results” below for more detail). A subset of 5 abstracts contained studies with comparison groups that did not match to groups 1–3 and were grouped into a fourth “other” category. As an added measure to limit the effects of inter-reviewer variability, categorized abstracts selected by the FSC (*n* = 67) underwent a second review by the EP to reassess for adherence to inclusion criteria. This iterative review resulted in agreement on all abstracts recommended by the FSC and EP with the exception of three abstracts. A final review conducted through our internal fidelity process determined that the abstracts in question did not fully adhere to inclusion criteria and were removed. Two of the removed abstracts were conference proceedings, one was determined not to have a defined comparison group (one additional abstract was subsequently found to be an oral abstract and removed). Sixty-three abstracts were advanced for full-text review. Further refining this set, group 1 had 3 articles that were removed as secondary analyses from 1 trial (the Maternal Opioid Treatment: Human Experimental Research (MOTHER) trial) [15]. While each of these articles had a distinct focus, they originated from the same dataset using the same clinical definition of NAS. Five articles were removed from group 2 that also represented secondary analyses stemming from the MOTHER trial.

### Full-text review

**Fig. 2:**
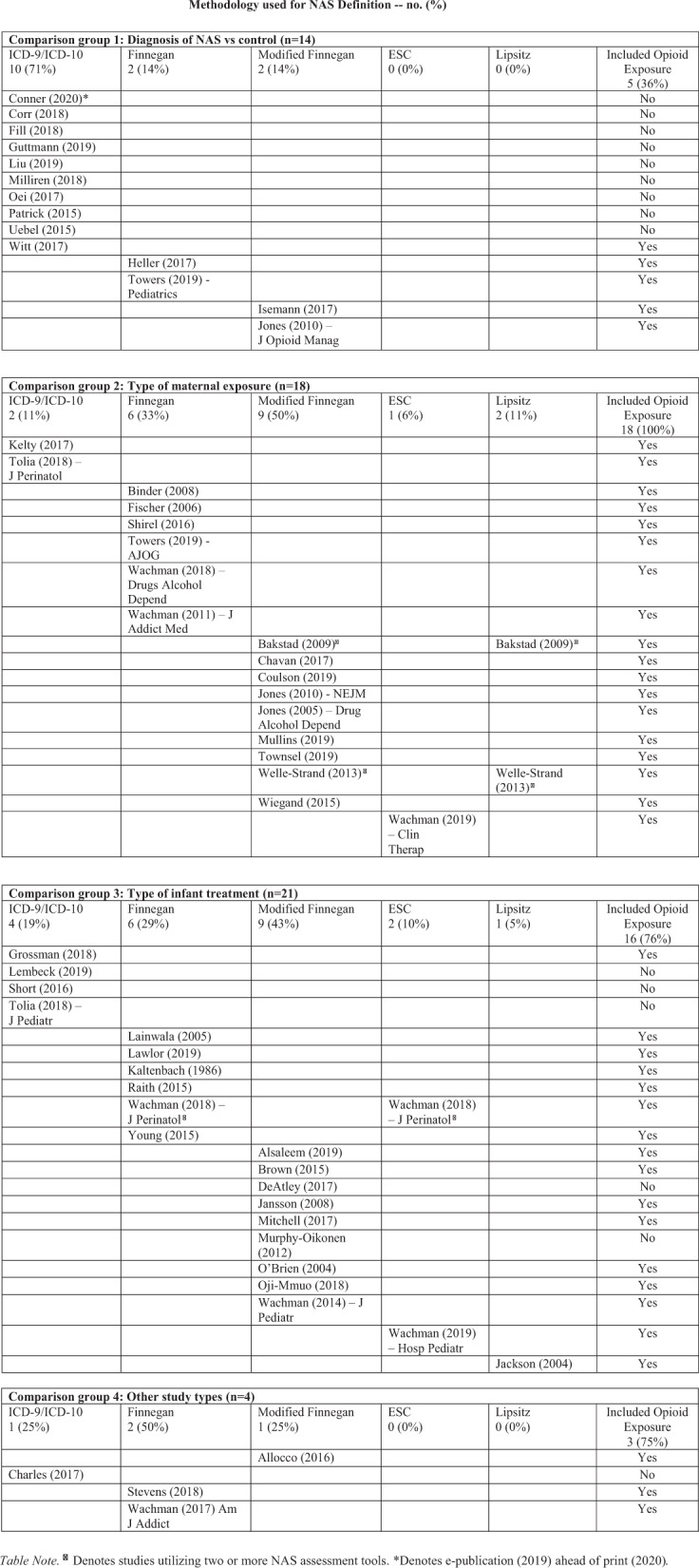
Evidence table summarizing abstracted findings from the full-text review of the 57 articles. Comparison groups were: (1) NAS vs control, (2) maternal exposure type, (3) infant treatment type, and (4) other. Studies within each category were then reviewed for consideration of opioid exposure of mother and/or infant as part of participant eligibility and/or inclusion criteria. ^**Δ**^Denotes studies citing two different methods to define NAS. *Denotes e-publication (2019) ahead of print (2020) retrieved by search parameters run in December 2019.

Collectively, 55 categorized abstracts were identified as adhering to inclusion criteria for this literature review. They underwent full-text review independently by two reviewers assessing two key characteristics: (1) method(s) that distinguished infants with a clinical diagnosis of NAS to those without and (2) opioid exposure for mother and infant. Independent review led to complete agreement with respect to key characteristics identified in each study. As a final measure of consistency, a confirmatory review of the full set of 888 abstracts was carried out as an iterative scan for abstracts eligible for inclusion; 2 additional abstracts were advanced for full-text review. An evidence table was constructed by abstracting pertinent information from the articles as a summary of findings from the full-text review. Overall, 57 articles (published from 1986 to 2019) adhered to the inclusion criteria for this literature review and were assessed for methodology used to define NAS (Fig. 2).

## Results

**Fig. 3:**
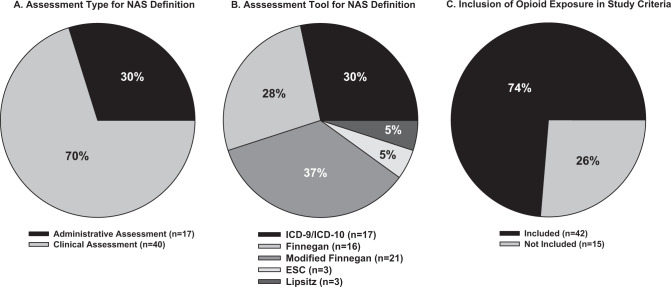
Pie charts summarizing abstracted findings collapsed across the three comparison groups. **A** Studies were evaluated for administrative vs clinical methodology cited to define NAS, described as: Administrative: International Classification of Diseases, Ninth or Tenth Revision (ICD-9/10), vs Clinical: the Finnegan NAS scoring system, modified versions of the Finnegan, Eat Sleep Console (ESC) and Lipsitz. **B** Breakdown of the total number of studies relying on each of the assessment tools. **C** Representation of the total number of studies considering opioid exposure of mother and/or infant as part of participant eligibility and/or inclusion criteria.

Focusing on individual study inclusion criteria revealed the use of both administrative (17/57; 30%) and clinical methods (40/57; 70%) to define NAS (Fig. 3). The administrative methods used were International Classification of Diseases, Ninth and Tenth Revision (ICD-9/10) codes and the clinical methods used were the Finnegan NAS scoring system, modified versions of the Finnegan, ESC, and Lipsitz. Of these methodologies, the modified Finnegan tool was cited most frequently to define NAS (37%) followed by ICD-9/10 coding (30%) and the Finnegan NAS scoring system (28%). The ESC (which has been proposed more recently) and Lipsitz assessment tools each represented only 5% of the total articles reviewed (note 3 abstracts used a combination of ≥2 tools). Although NAS scoring and assessment tools (along with the requirement for pharmacologic treatment if scores were high enough) were not the main focus of this literature search, a majority of studies described these items as key criteria used to define NAS.

Most articles included in our review fell into 3 different categories of participant comparison groups: (1) NAS vs control, (2) maternal exposure type, (3) infant treatment type, and a fourth “other” category of articles that did not sort into groups 1–3. For group 1, 71% (10/14) of studies cited use of administrative coding data as a determinant for the NAS definition. This is traditionally seen with insurance claims data and/or other large billing data sources. Studies within this collective group examined clinical, developmental, socioeconomic, demographic, and healthcare utilization characteristics between children diagnosed with NAS compared to those who were not. The majority of studies in group 1 (64%; 9/14) did not include opioid exposure for mother or infant as part of their inclusion criteria. For group 2, 100% (18/18) of studies emphasized opioid exposure as part of their inclusion criteria. Studies within this group collectively examined neonatal outcomes in the setting of maternal exposure to opioids and/or maternal medication for opioid use disorder. For group 3, most studies 81% (17/21) cited use of clinical methodologies in defining NAS, including opioid exposure in 76% (16/21). These studies collectively examined neonatal outcomes in the setting of different treatment options for NAS, including supportive non-pharmacologic and pharmacotherapeutic approaches. For group 4, 2 articles examined sex differences in infants diagnosed with NAS, 1 examined allelic differences and NAS outcomes, and 1 study investigated NAS in preterm vs term infants. Three of the 4 (75%) studies in group 4 included opioid exposure in their participant inclusion criteria.

Specific consideration was given as to whether individual trials included opioid exposure as a crucial component of the study’s inclusion criteria for defining NAS (Fig. 3). While 74% (42/57) of studies identified by this review included opioid exposure as an integral part of their participant eligibility and/or inclusion criteria for mothers, infants, and/or the mother–infant dyad, 26% (15/57) did not. This was unexpected as opioid exposure is traditionally considered an essential element of the definition of NAS.

## Discussion

This expert literature review illustrates specific methodologies most frequently cited to define NAS in clinical studies of mothers, infants, and mother–infant dyads. Our findings raise a number of concerns about inconsistencies in the way NAS has been defined. A majority of studies cite a modified version of the Finnegan NAS scoring system or the use of administrative coding data as the preferred method to define NAS. However, both approaches present significant challenges.

First, with the use of scoring tools, the diagnosis is frequently dependent on the infant’s score and linked to the infant’s need for pharmacologic therapy for NAS. Diagnosis is therefore limited to infants meeting threshold scoring for therapeutic intervention. However, for opioid- or polysubstance-exposed infants not meeting minimum scoring criteria for the initiation of pharmacotherapy for NAS, the clinical diagnosis may vary depending on the preferences of providers and/or facilities [16], with some using the diagnosis of NAS to describe the clinical features seen in these infants and some not. Importantly, scoring assessments may also be influenced by inter-rater variability and are inherently subjective [17]. Agreement on 90% of the scored elements is acceptable between raters using the original Finnegan NAS scoring system [18] and achievement of this benchmark requires and relies on proper provider training and education. Without it, incorrect use of the scoring tool may result in errors in treatment [19] and likewise could be similarly problematic for the linked question of NAS diagnosis.

Second, the use of medical claims data may reflect inter-coder differences in the assignment of diagnosis and billing codes for NAS. This method may also be subjective and not necessarily directly associated with the infant’s clinical presentation. Though administrative data has been independently validated in identifying a clinical diagnosis of NAS with high positive predictive value [20], it often serves as a source of data for NAS surveillance and is prone to upstream variabilities in clinical diagnosis. In addition, a recent review examining NAS clinical and surveillance definitions at the state level revealed that only 3 of the 44 states participating in NAS-related data activities had both a specified clinical and surveillance definition in place [21]. A subset of the remaining states had one or the other but not both. Since this report, there have been opportunities for states to transition to implementation of the CSTE surveillance definition, but the need for a specified clinical definition remains.

Third, it was interesting that not all selected studies included maternal–infant opioid exposure as an essential component of the NAS definition. As noted above, NAS is most commonly conceptualized as withdrawal from opioids in exposed infants, but many studies may define NAS as withdrawal from a variety of exposures, not exclusively opioids. Other substance exposures beyond opioid use can affect NAS expression, including the demonstration of withdrawal signs/symptoms and their severity and/or pharmacotherapy decisions for the infant. In any case, the lack of inclusion of opioid exposure as an essential component in the diagnosis of NAS appears problematic given the prominent role of in utero opioid exposure—though often in the context of other exposures—in the development of NAS [13]. Most noteworthy, this key knowledge gap highlights the critical need for addressing the definition of NAS at a point fundamentally upstream of the use of a scoring tool or administrative coding—namely, at the bedside at a clinical diagnostic level. It reinforces the importance of a standardized clinical definition for NAS as an essential step in advancing our discussions. This is crucial not only for clinical trials of new and existing therapeutics but also for improving downstream needs assessment and care for the appropriately identified mother–infant dyad.

While our approach carries the limitations of a non-systematic review, it offers a conceptual summary of the limited number of available randomized control and cohort trials and the criteria they used to investigate various key outcomes for the mother–infant dyad. At least three limitations can be noted. Our emphasis on study designs with comparison groups may have excluded more descriptive literature that could also offer insight on how NAS has been historically defined. Likewise, some of the studies selected for this literature review demonstrated low population size, correlational design, and may be subject to underlying biases. Additionally, though the keyword search was informed by evidence-based guidelines and an environmental scan, it may have missed articles not captured by our focused search strategy and potentially retrievable by different search methods.

As the foundational step of an overarching RAM focusing on the clinical definition of NAS, the findings from this RAM literature review of comparative clinical studies will inform broader discussions on challenges with existing methods used to define NAS and important inconsistencies and gaps that need to be addressed. A key next step to closing fundamental knowledge gaps will be to develop standard clinical criteria for the diagnosis of NAS that are both practical and broadly applicable to clinical practice. The core consideration for criteria would be grounded in the infant’s clinical signs of withdrawal independent of the need for pharmacologic treatment. A diagnosis of NAS in an infant who does not meet criteria for medication to alleviate symptoms may allow the application of targeted non-pharmacologic therapies to address NAS-related difficulties. Other important elements would include integration of (1) opioid and other substance exposures consistent with signs of neonatal withdrawal, (2) available maternal data, in a manner that does not contribute to further stigmatization of mothers with history of opioid/substance use, and (3) maternal–infant toxicology, when available, to document the exposure(s).

## Conclusion

Addressing the current gap in knowledge and providing a standard clinical definition for NAS would lay the foundation for informing public health policy; improve the accuracy and reproducibility of NAS surveillance, administrative claims, clinical records and scientific reports; and can enhance needs assessment and care for mother–infant dyads affected by opioid exposure in the short and long term.
